# Allelic heterogeneity and abnormal vesicle recycling in *PLAA*-related neurodevelopmental disorders

**DOI:** 10.3389/fnmol.2024.1268013

**Published:** 2024-04-08

**Authors:** Michele Iacomino, Nadia Houerbi, Sara Fortuna, Jennifer Howe, Shan Li, Giovanna Scorrano, Antonella Riva, Kai-Wen Cheng, Mandy Steiman, Iskra Peltekova, Afiqah Yusuf, Simona Baldassari, Serena Tamburro, Paolo Scudieri, Ilaria Musante, Armando Di Ludovico, Sara Guerrisi, Ganna Balagura, Antonio Corsello, Stephanie Efthymiou, David Murphy, Paolo Uva, Alberto Verrotti, Chiara Fiorillo, Maurizio Delvecchio, Andrea Accogli, Mayada Elsabbagh, Henry Houlden, Stephen W. Scherer, Pasquale Striano, Federico Zara, Tsui-Fen Chou, Vincenzo Salpietro

**Affiliations:** ^1^Unit of Medical Genetics, IRCCS Istituto Giannina Gaslini, Genoa, Italy; ^2^Division of Biology and Biological Engineering, California Institute of Technology, Pasadena, CA, United States; ^3^Department of Chemical and Pharmaceutical Sciences, University of Trieste, Trieste, Italy; ^4^Genetics and Genome Biology, The Hospital for Sick Children, Toronto, ON, Canada; ^5^The Centre for Applied Genomics, The Hospital for Sick Children, Toronto, ON, Canada; ^6^Department of Pediatrics, Sant'Annunziata Hospital, University “G. D'Annunzio”, Chieti, Italy; ^7^Department of Biotechnological and Applied Clinical Sciences, University of L'Aquila, L'Aquila, Italy; ^8^Department of Neurosciences, Rehabilitation, Ophthalmology, Genetics, Maternal and Child Health (DiNOGMI), University of Genoa, Genoa, Italy; ^9^Montreal Neurological Institute-Hospital, Azrieli Centre for Autism Research, McGill University, Montreal, QC, Canada; ^10^McGill University Health Centre, McGill University, Montreal, QC, Canada; ^11^Pediatric Neurology and Muscular Diseases Unit, IRCCS Istituto Giannina Gaslini, Genoa, Italy; ^12^Department of Clinical Sciences and Community Health, University of Milan, Milan, Italy; ^13^Department of Neuromuscular Diseases, UCL Institute of Neurology, London, United Kingdom; ^14^Clinical Bioinformatics Unit, IRCCS Istituto Giannina Gaslini, Genoa, Italy; ^15^Department of Pediatrics, University of Perugia, Perugia, Italy; ^16^Division of Medical Genetics, Department of Specialized Medicine, McGill University, Montreal, QC, Canada; ^17^Department of Molecular Genetics, University of Toronto, Toronto, ON, Canada; ^18^McLaughlin Centre, University of Toronto, Toronto, ON, Canada; ^19^Proteome Exploration Laboratory, Beckman Institute, California Institute of Technology, Pasadena, CA, United States

**Keywords:** *PLAA* gene, *de novo* variants, neurodevelopmental disorders, synaptic transmission, SNAREopathies, developmental regression

## Abstract

The human *PLAA* gene encodes Phospholipase-A2-Activating-Protein (PLAA) involved in trafficking of membrane proteins. Through its PUL domain (PLAP, Ufd3p, and Lub1p), PLAA interacts with p97/VCP modulating synaptic vesicles recycling. Although few families carrying biallelic *PLAA* variants were reported with progressive neurodegeneration, consequences of monoallelic *PLAA* variants have not been elucidated. Using exome or genome sequencing we identified *PLAA de-novo* missense variants, affecting conserved residues within the PUL domain, in children affected with neurodevelopmental disorders (NDDs), including psychomotor regression, intellectual disability (ID) and autism spectrum disorders (ASDs). Computational and *in-vitro* studies of the identified variants revealed abnormal chain arrangements at C-terminal and reduced PLAA-p97/VCP interaction, respectively. These findings expand both allelic and phenotypic heterogeneity associated to *PLAA*-related neurological disorders, highlighting perturbed vesicle recycling as a potential disease mechanism in NDDs due to genetic defects of PLAA.

## 1 Introduction

Genetic brain developmental disorders with associated psychomotor delay and/or regression include a broad variety of monogenic conditions with expanding clinical differential diagnosis, genetic heterogeneity and associated disease mechanisms (Salpietro et al., 2017; Zollo et al., 2017; Niccolini et al., 2018; Ghosh et al., 2021). Also in this era of next-generation sequencing (NGS), the etiology and disease mechanisms underlying neurodevelopmental impairment remains undetermined in a large proportion of cases (Salpietro et al., 2018a,b; Neuray et al., 2020; Epi25 Collaborative, 2021). Defining the full spectrum of disease-causing molecular pathways underlying neurodevelopmental disorders (NDDs) is pivotal in diagnosing patients with developmental delay or regression and to assess potential personalized strategies for the follow-up and the management of the affected children (Pavlidou et al., 2016; Ruggieri et al., 2016a; Iacomino et al., 2020). NDDs encompass a range of frequently co-existing conditions including developmental delay (DD), intellectual disability (ID), and autism spectrum disorders (ASDs). ASDs are neurodevelopmental conditions characterized by impaired social communication, repetitive patterns of behavior, interests and activities, as well as sensory processing anomalies (American Psychiatric Association, 2013). In most children diagnosed with ASDs, anomalies in speech and social communication are usually observed within the 1st year of life, but some can present an apparently typical development in early infancy followed by an history of neurodevelopmental regression and the loss of previously established skills (Ozonoff et al., 2008, 2018; Pearson et al., 2018). Etiological factors underlying neurodevelopmental delay/regression in the context of NDDs are highly heterogeneous and likely include both genetic and environmental causes. Molecular studies dissecting the complex genetic architecture of ASD-associated single gene disorders highlight frequent deleterious variants in genes often implicated in important roles of transcriptional and synapse regulation, including vesicle release or recycling as well as post-synaptic transmission (Salpietro et al., 2019a,b). However, the identified genetic causes underlying ASDs have explained so far only a small percentage of risk, but given the high heritability, there is a great deal to do. In this study, we report on children affected with neurodevelopmental impairment or regression found to carry *de novo* variants in the *PLAA* gene, encoding the Phospholipase-A2-Activating-Protein. This expands both the phenotypic and allelic heterogeneity associated with *PLAA*-related neurological disorders as the gene has only been implicated so far in ultra-rare autosomal recessive neurodegenerative disorders associated to microcephaly, leukodystrophy and early lethality. In addition, our functional studies implicate that the abnormal Plaa/p97 interaction and the resulting abnormal vesicle recycling and brain development processes are a consequence of the changes to the PUL domain due to *de novo PLAA* variants.

## 2 Materials and methods

### 2.1 Screening genomic datasets of NDDs for *PLAA* variants

We screened for biallelic and/or *de novo* variants in *PLAA* genomic datasets part of the SYNaPS study group consortia (which contains exome sequencing data from ~8,000 individuals affected with heterogeneous brain developmental disorders; http://neurogenetics.co.uk/synaptopathies-synaps-2) and the MSSNG database for autism researchers (https://research.mss.ng). Furthermore, we interrogated publicly available databases, including DECIPHER/DDD (https://www.deciphergenomics.org), LOVD (https://www.lovd.nl) and the Matchmaker Exchange platform (Azzariti and Hamosh, 2020). Written informed consent for families who underwent whole exome sequencing (WES) and whole genome sequencing (WGS) was obtained under protocols approved by local institutional review boards. The study has been approved by the ethics committee of Children's Hospital “Giannina Gaslini” in Genova (Italy) and the other participating centers.

### 2.2 Genetic analysis and variant interpretation

Written informed consent for genetic sequencing was obtained by the parents or legal guardians of affected individuals. NGS techniques (trio WES in Individual 1 and WGS in Individual 2) were performed as previously described (Salpietro et al., 2018c; Efthymiou et al., 2019). Libraries were prepared from parent and patient DNA, and were sequenced on NovaSeq 6,000 Illumina sequencers. Sequencing data were processed using commercial tools for the execution of the GATK Best Practices pipeline. Variant annotation was performed using analytical pipelines that include publicly available tools and custom scripts (Ensembl-VEP v.100). Exonic and donor/acceptor splicing variants were considered for further analysis. Priority was given to rare variants that were present at <1% frequency in public databases, such as 1,000 Genomes Project, NHLBI Exome Variant Server and the Genome Aggregation Database (GnomAD) and had a genomic evolutionary rate profiling (GERP) score >2. Synonymous variants were not considered. Candidate *de novo*, biallelic and X-linked hemizygous coding variants were filtered according to genetic criteria for their phenotypic and biological impact. Validation, parental origin of the resulting variants and family segregation studies were performed by traditional Sanger sequencing.

### 2.3 Homology modeling and molecular dynamics simulations

Initial monomeric tridimensional structures for the PUL domain of PLAP were generated through the automated homology-modeling server SWISS-MODEL (Waterhouse et al., 2018) by employing the PLAA crystallographic structure 3EBB as a template (PDB ID 3EBB; aa. 531–795, chain A; Lomize et al., 2012). Each model was then placed in a cubic box with a water layer of 1.0 nm, neutralized with Na+ and/or Cl- ions, and minimized. The steepest descent minimization stopped either when the maximum force was lower than 1,000.0 kJ/mol/nm or when 50,000 minimization steps were performed with 0.005 kJ/mol energy step size, Verlet cutoff scheme, short-range electrostatic cut-off and Van der Waals cut-off of 1.0 nm. AMBER99SB-ILDN force field, tip3p water, and periodic boundary conditions were employed. Nose-Hoover thermostat (NVT) and Nose-Hoover thermostat (NPT) equilibrations were performed for 100 ps by restraining the protein backbone, followed by 500 ns long NPT production runs at 300 K. The iteration time step was set to 2 fs with the Verlet integrator and LINCS constraint. All the simulations and their analysis were run as implemented in the Gromacs package v. 2020.319. Radius of gyration, RMSD, and RMSF have been calculated from configurations sampled every 0.5 ns. Simulations were run on M100 (CINECA, Italy). The electrostatic surfaces have been calculated according to the Adaptive Poisson-Boltzmann Solver method, APBS-PDB2PQR software suite (https://server.poissonboltzmann.org).

### 2.4 Molecular cloning and mutagenesis

The construct containing the cDNA of wild type full-length human PLAA (residues 1–795) was obtained from UCLA molecular screening shared resource. A Myc-tag followed by a TEV cut site was introduced at the N-terminal of full-length PLAA using two overlapping forward primers containing the Myc-TEV sequence. The c.2383C>A; p.Leu795Met mutation was introduced using a reverse primer containing the mutation. The WT and p.Leu795Met mutant PLAA cDNAs were PCR-amplified and subcloned into linearized pcDNA3.3 vector using XbaI and BamHI restriction enzymes (New England Biolabs). The PCR products were cloned into the vector using the Gibson assembly method (New England Biolabs), which does not necessitate the use of restriction enzymes. The c.1826T>C; p.Ile609Thr mutation was introduced into the wild type Myc-TEVPLAA construct using site directed mutagenesis with the QuikChange XL kit (Agilent). All constructs were sequenced with Sanger sequencing. All primers used are listed in the Supplementary material.

### 2.5 Cell maintenance and transfection

HEK-293T cell line (CRL-3216) was purchased from ATCC. Cells were cultured in DMEM media (Sigma-Aldrich) supplemented with 10% fetal bovine serum (FBS, Atlanta Biologicals), 100 μg/mL streptomycin, and 100 U/mL of penicillin (Lonza). Cells were maintained at 37°C with 5% CO_2_. For immunoprecipitation experiments, 10 million cells were seeded in 15 mm plates. The following day, cells were transfected with 25 μg of plasmid in triplicates using BioT reagent (Bioland Scientific LLC) and were harvested 24 h post transfection.

### 2.6 Immunoprecipitation

Cell pellets were washed with cold PBS and harvested according to standard procedures. All of the buffers used were made using OptimaTM LC/MS grade water (Fisher). Cells were lysed in lysis buffer [20 mM HEPES pH 7.5, 100 mM NaCl, 0.2% n-dodecyl-β-D-maltoside, 20 μM MG132, 1 mM N-ethylmaleimide, one tablet of complete protease inhibitor (Roche, Germany)] for 10 min on a rotator at 4°C. Lysates were cleared by centrifugation for 15 min at 16,600 g at 4°C and supernatant was transferred to a new tube. Protein concentrations were determined using Bradford reagent (Bio-rad). For each IP, Myc-beads (50 μl, Thermo Fisher) were washed twice with 1 ml lysis buffer. Clear cell lysate was incubated with Myc-beads at 4°C for 2 h. Beads were washed with 1 ml lysis buffer three times and 100 mM Tris buffer (pH 8.5) twice. Proteins were eluted with 100 μl of 10 M urea in 100 mM Tris buffer (pH 8.5) by incubating at 37°C for 15 min and then samples were spun down using Bio-rad micro bio-spin chromatography column to collect the eluate. 10 μl of eluate was saved for Western blot analysis and the rest was further processed for Mass Spectrometry.

### 2.7 Western blot analysis

For western blot analysis, 4x Laemmli sample buffer (Bio-Rad, cat# 161-0774) containing 0.1 M DTT (Cytiva, cat#17-1318-02) was mixed with the samples and heated for 5 min at 95°C. Comparable amounts (~10 μg) of protein samples were loaded onto a 4%-20% SDS-PAGE gel and transferred to a nitrocellulose membrane. The membrane was then blocked with 5% milk for 1 h and probed with anti-VCP antibody at 1:3000 (Thermo Fisher Scientific, MA3-004) for detecting p97, anti-MYC antibody at 1:1000 (EMD Millipore, 05–724) for detecting MYC-PLAA, anti-SAKS1 antibody at 1:1000 (Proteintech, 16135-1-AP) for detecting SAKS1 protein, and anti-GAPDH antibody at 1:5000 (Cell Signaling Technologies, 21,185) for detecting GAPDH. Primary antibodies were detected using HRP-labeled goat anti-mouse or anti-rabbit antibodies (Bio-Rad). Blots were developed using Immobilon Western Chemiluminescent HRP Substrate (Millipore) and visualized using ChemiDoc MP Imaging System (Bio-Rad). Blot densities were analyzed using Image Lab 6.0.1 software (Bio-Rad).

### 2.8 Digestion and LC-MS/MS

The IP eluted samples were digested as follows: (1) 25 μL 100 mM Tris buffer (pH 8.5) and 1 μL 0.5 M TCEP (Thermo) was added and samples were incubated at 37°C for 20 min on shaker; (2) 3 μL 0.5 M 2-chloroacetamide (MP Biomedicals) with incubation at 37°C for 15 min on shaker; (3) 2 μL 0.1 μg/μL Lys-C (FUJIFILM) with incubation at 37°C for 4 h on shaker; (4) 375 μL 100 mM Tris buffer (pH 8.5), 5 μL 100 mM CaCl_2_, and 3 μL 0.1 μg/μL Trypsin (Thermo) with incubate at 37°C for 20 h with shaking. The digested samples were acidified with 20% TFA (Thermo), desalted using Pierce C18 spin columns (Thermo), and dried using in a vacuum centrifuge. Prior to running mass spec, samples were dissolved in 15 μL 0.2% FA water solution. The LC-MS/MS experiments were performed by loading 5 μL sample onto EASY-nLC 1200 (Thermo) connected with Orbitrap Eclipse Tribrid mass spectrometer (Thermo). Peptides were separated on an Aurora UHPLC Column with a flow rate of 350 nL/min and a total duration time of 75 min following the gradient composed of 2–6% Solvent B for 3.5 min, 6–25% B for 41 min, 25–40% B for 15 min, 40–98% B for 1 min, and 98% B for 14 min. Solvent A consists of 97.8% H_2_O, 2% ACN, and 0.2% formic acid; solvent B consists of 19.8% H_2_O, 80% ACN, and 0.2% formic acid. MS1 scans were acquired with a range of 350–1,600 m/z in the Orbitrap at 120 k resolution. MS2 scans were acquired with HCD activation type in the Ion Trap. Method modification and data collection were performed by using Xcalibur software (Thermo). Proteomic analysis was performed with Proteome Discoverer 2.4 (Thermo Scientific) software.

## 3 Results

### 3.1 Identification of *PLAA* variants

**Figure 1:**
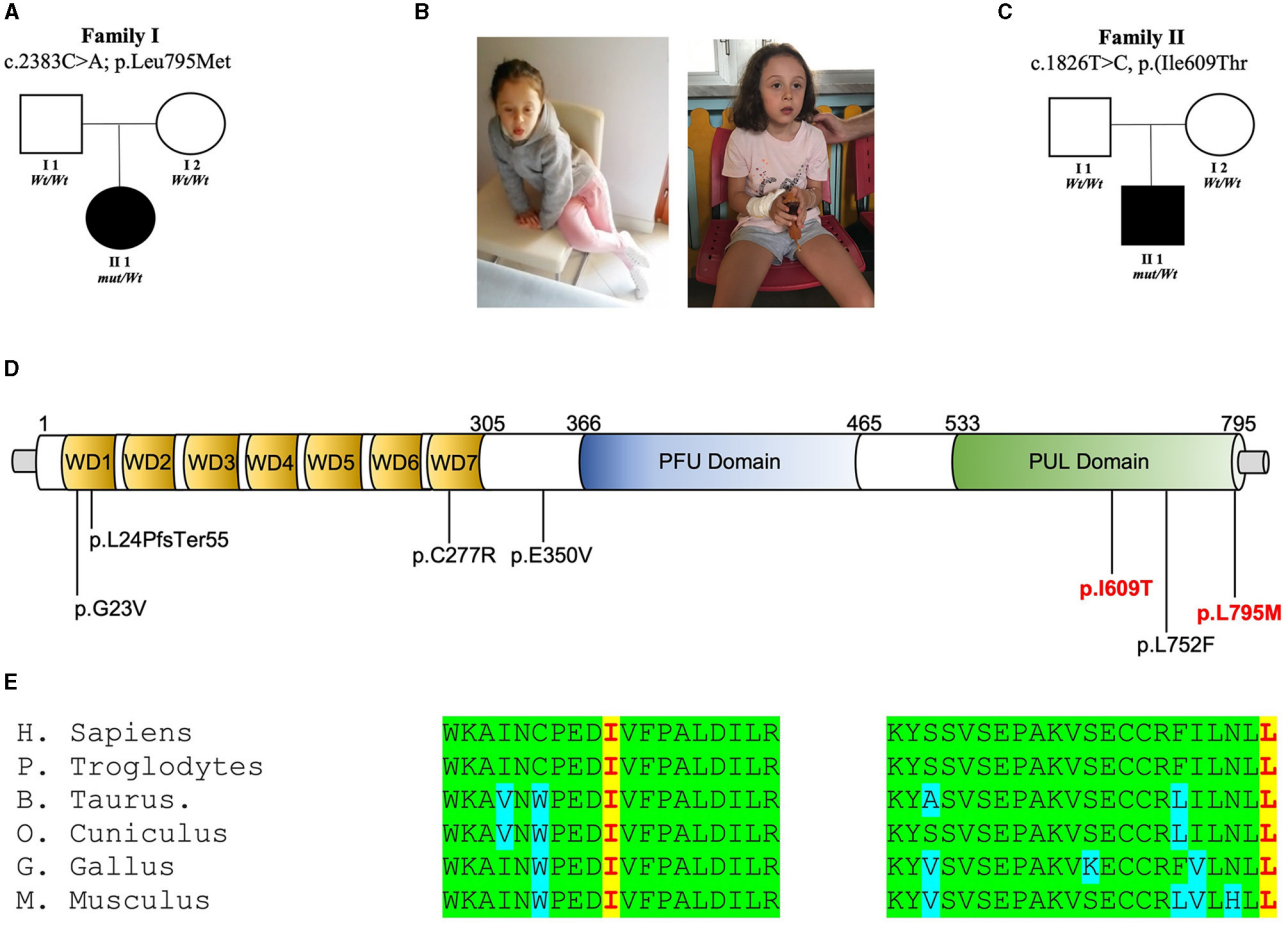
*PLAA* intragenic *de novo* variants identified in this study. **(A)** Family 1 pedigree: squares and circles represent males and females, respectively. Closed symbols denote affected individuals. Open symbols denote unaffected individuals. Electropherogram showing heterozygous mutation c.C2383A [p.L795M] and wild-type (wt) sequences. **(B)** Photos of the patients. **(C)** Family 2 pedigree: squares and circles represent males and females, respectively. Closed symbols denote affected individuals. Open symbols denote unaffected individuals. Electropherogram showing heterozygous mutation c.T1826C [p.I609T] and wild-type (wt) sequences. **(D)** Representation of the wild-type (Leu795) PLAP vs. the mutant (Met795) and representation of the wild-type (Ile609) PLAP vs. the mutant (Thr609). **(E)** Multiple alignment showing PLAA protein complete conservation across species alignment for p.Leu795 and Ile609 aminoacids in red squares.

Two novel *de novo* variants in *PLAA* [NM_001031689.3: c.1826T>C, p.(Ile609Thr); c.2383C>A, p.(Leu795Met)] were found from the screening of the MSSNG and SYNAPS databases, respectively. In these families (Figures 1A–C), no pathogenic or candidate variant in any of the known disease genes were found, and WES/WGS led to the identification of these *de novo* non-synonymous variants replacing highly conserved residues within the PUL domain of PLAA (Figures 1D, E).

### 3.2 Clinical phenotype associated with *de novo* variants in *PLAA*

#### 3.2.1 Individual 1

The patient was the first child of a healthy, non-consanguineous Italian couple (Figures 1A, B). There was no family history of neurodevelopmental disease in the family. She was born full term and her body measurements were all within normal ranges. During early infancy, the girl exhibited normal development. She acquired some clear words at 15 months of age. Her motor developmental milestones were normal in the first 2 years of life. Occipitofrontal circumference (OFC) was always within normal ranges and she has no distinctive facial features. Since the age of 3.5 years, she showed signs of developmental regression. She lost her verbal ability and skills in activities of daily living. Moreover, she lacked eye contact. At 4 years of age, she became obsessed with spinning parts of a particular toy. She also displayed some hand stereotypies (e.g., hand wringing, hand clapping), resembling individuals affected with Rett syndrome. These signs were transient and not observed a few months later. Electroencephalogram and brain magnetic resonance imaging were unremarkable. She had a Leiter IQ score of 80. Based on the results obtained at the parental interview and the disturbances observed in social skills and communication and the CBCL and HFA tests, she was diagnosed with ASD. At the current age of 5 years, the father reported some improvements in regard to her speech and communication abilities with improved interactions with other children. In this family, trio-based WES was performed and revealed a *de novo* non-synonymous variant in *PLAA* [NM_001031689.3: c.2383C>A, p.(Leu795Met)].

#### 3.2.2 Individual 2

The patient was the child of a healthy, non-consanguineous Canadian couple (Figure 1C). There was no family history of neurodevelopmental disease in the family. He was born full term by spontaneous delivery and his weight and length were within normal ranges. His occipitofrontal circumference (OFC) at birth was 34 cm (10–25th centile). During the 1st year of life, the boy exhibited normal neurological development. Since the age of 15 months the mother noticed some regression in language and communication abilities. He was diagnosed with ASD at the age of 2 years. At 6 years old, based on the results obtained at the parental interview of the ADI-R and the observations of the ADOS, he continued to exhibit difficulties that are consistent with a diagnosis of autism but showed progresses in social skills, play and communication. At that time, the Merrill-Palmer-Revised Cognitive test revealed a developmental functioning at an equivalent age of about 30 months (<1th). At his last assessment at the age of 9 years, speech abilities improved and his mother reported his ability to communicate in full sentences and the ability to play symbolic games with other children. The patient was identified by screening the MSSNG Database for Autism researchers. Trio WGS was performed at the Hospital for Sick Children (SickKids) in Toronto (Canada) and led to the identification of a *de novo* variant in *PLAA* [NM_001031689.3: c.1826T>C, p.(Ile609Thr)].

### 3.3 Computational dynamic simulations

**Figure 2:**
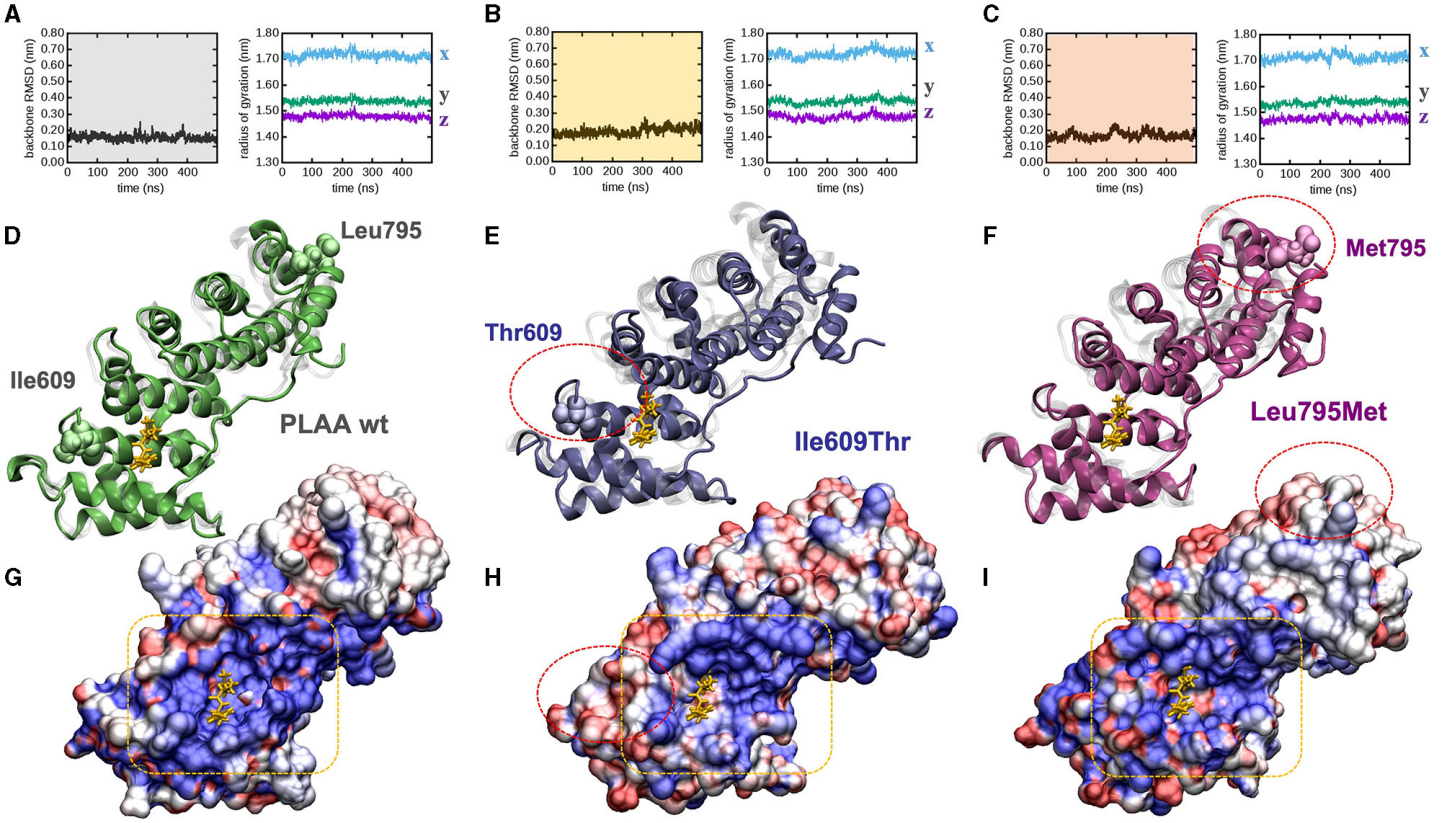
PLAA molecular modeling and dynamic simulations. **(A–C)** Molecular dynamics trajectories analysis: backbone root mean squared deviation **(left)** and x, y, x, components of the backbone radius of gyration **(right)** for **(A)** wt, **(B)** Ile609Thr, and **(C)** Leu795Met. **(D, E)** End-simulation configuration for **(D)** wt, **(E)** Ile609Thr, and **(F)** Leu795Met, the configurations are superposed to the crystallographic structure 3EBB, chain A, where PLAA (shaded in gray) is bound to a the p97 fragment (highlighted in yellow). **(G–I)** Solvent accessible surface area of **(G)** wt, **(H)** Ile609Thr, and **(I)** Leu795Met, also in this case the configurations are superposed to the crystallographic structure 3EBB and the p97 fragment is highlighted (yellow), the surfaces are colored according to the calculated electrostatic potential at each surface point: positive (blue), negative (red), and neutral/hydrophobic (white). For ease of comparison among panels red circles highlight the mutations in **(E–I)**, and a yellow box highlights the PLAA positively charged groove **(G–I)**. All configurations are snapshots taken at 500 ns. Water molecules have been omitted for ease of interpretation.

To explore whether and how the missense mutations affect the protein fold, we modeled each mutant by homology from the experimentally determined wild type x-ray structure, and explored their behavior in time by means of atomistic molecular dynamics (MD) simulations in full water solvent. Interestingly all mutants are fairly stable along the 500 ns of simulated time: backbone atoms did not displace more than 0.3 nm from their initial configuration (as evinced by their root mean squared deviation, or RMSD, Figures 2A–C, left panels), nor the proteins backbone changed sensibly their overall shape and size along the simulated time (as evinced by the stability of the radius of gyration Figures 2A–C, right panels). After 500 ns, the protein fold observed in the PLAA-wt crystallographic structure was still present in all mutants (Figures 2D–F). PLAA-wt has been observed to be composed by six Armadillo repeats connected by flexible linkers (Lomize et al., 2012). These are still present in all mutants after 500 ns (Figures 2D–F). In PLAA-wt, the third helix of each repeat is known to form a positively charged groove shown to interact with a p97 fragment (Figure 2G). In p.Ile609Thr, besides removing one residue possibly directly interacting with p97, the substitution of a hydrophobic residue with a polar one lead to a reduction of the groove positive charge and the creation of hydrophobic areas which, in turn, cause a loss of affinity to p97 (Figure 2H) based on computational evaluations (Wang et al., 2020). The p.Leu795Met variant does not seem to affect the protein fold but it may affect the functioning of the protein due to long distance effects. Indeed, Met 795 introduces a perturbation affecting the groove side chain arrangements (Figure 2I) which, while changing its polarization to a lesser extent than that observed in Ile609Thr, changes its shape by reducing its size. These combined effects are expected to compromise its binding affinity for p97.

### 3.4 IP/Mass Spectrometry shows that p.Leu795Met and Ile609Thr variants affect the PLAA interactome

**Figure 3:**
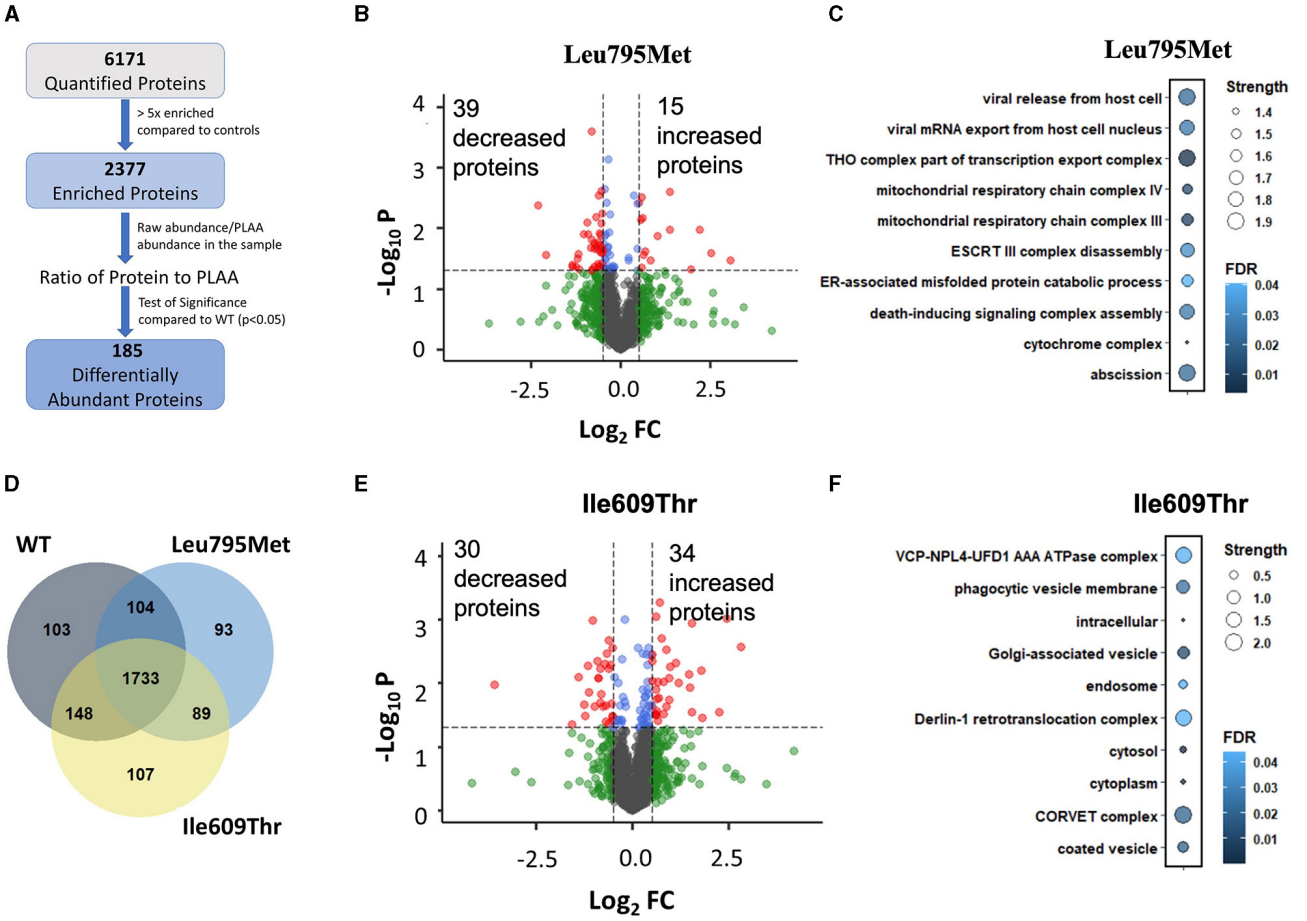
Mass Spectrometry of wild type, p. Ile609Thr and p. Leu795Met Immunoprecipitation. **(A)** A flow-through of the mass spectrometry data analysis steps. Briefly, proteins were first filtered based on enrichment relative to controls (>5x), then for each protein, a two-tailed student's *t*-test was used to compare each of the mutants to the wild type. **(B–E)** Volcano plot showing the differentially pulled down proteins in the Leu795Met and Ile609Thr samples compared to wild type. Cutoff lines are set to *p* = 0.05 and |Log2FC|>0.5. **(D)** Venn diagram of the remaining proteins after filtering, i.e., proteins which are >5x enriched in each of the IP conditions compared to untransfected controls. **(C–F)** Gene ontology enrichment analysis. Analysis was performed through STRING website. All terms (including BP, CC, and MF) were combined and ranked in descending order based on Strength. The top 10 terms were kept and displayed.

To investigate if the two *de novo* PLAA variants affect its interactome, we generated wild type and mutant PLAA plasmids with an N-terminus Myc tag. We then overexpressed the PLAA constructs in HEK293T cells and pulled down PLAA using Myc beads. The eluted samples were digested and run through LC-MS/MS. Our Mass Spectrometry data quantified a total of 6171 proteins. To filter out non-specific binding, we used a cut off of 5-fold enrichment compared to the control samples. Following this, 2,377 proteins remained (Figure 3A). Of those proteins, 1,733 (73%) were enriched in all 3 IP conditions while 103, 93, and 107 were only enriched in WT, p.Leu795Met and p.Ile609Thr, respectively (Figure 3B). This suggests that both mutations do not abolish the interactions of PLAA with most of its targets. In order to identify proteins that are differentially pulled down by wild type vs. mutant PLAA, we divided the raw protein abundance of each protein by that of PLAA in the sample and compared this ratio in the WT and mutant samples. There were a total of 90 differentially abundant proteins in the p.Leu795Met mutant samples compared to WT (*p* < 0.05). Of them, only 54 had a |log2FC|>0.5 (Figure 3C). For the p.Ile609Thr mutant, there was in total 109 differentially abundant proteins of which 64 had |log2FC|>0.5. However, only 14 proteins were differentially abundant in both mutants. To determine if the differentially abundant proteins belong to similar cellular pathways and complexes, we performed Gene Ontology analysis on all significant proteins (Figure 3D). Enrichment analysis of the differentially abundant proteins in the IP of Leu795Met reveals proteins affected are related to vesicle formation and transport as well as mitochondrial proteins (Figure 3E). Enrichment analysis of differentially abundant proteins in the IP of p.Ile609Thr reveals proteins associated with protein transport and ERAD (Figure 3F).

### 3.5 PLAA binding to VCP/p97 is reduced in both *PLAA* mutants

**Figure 4:**
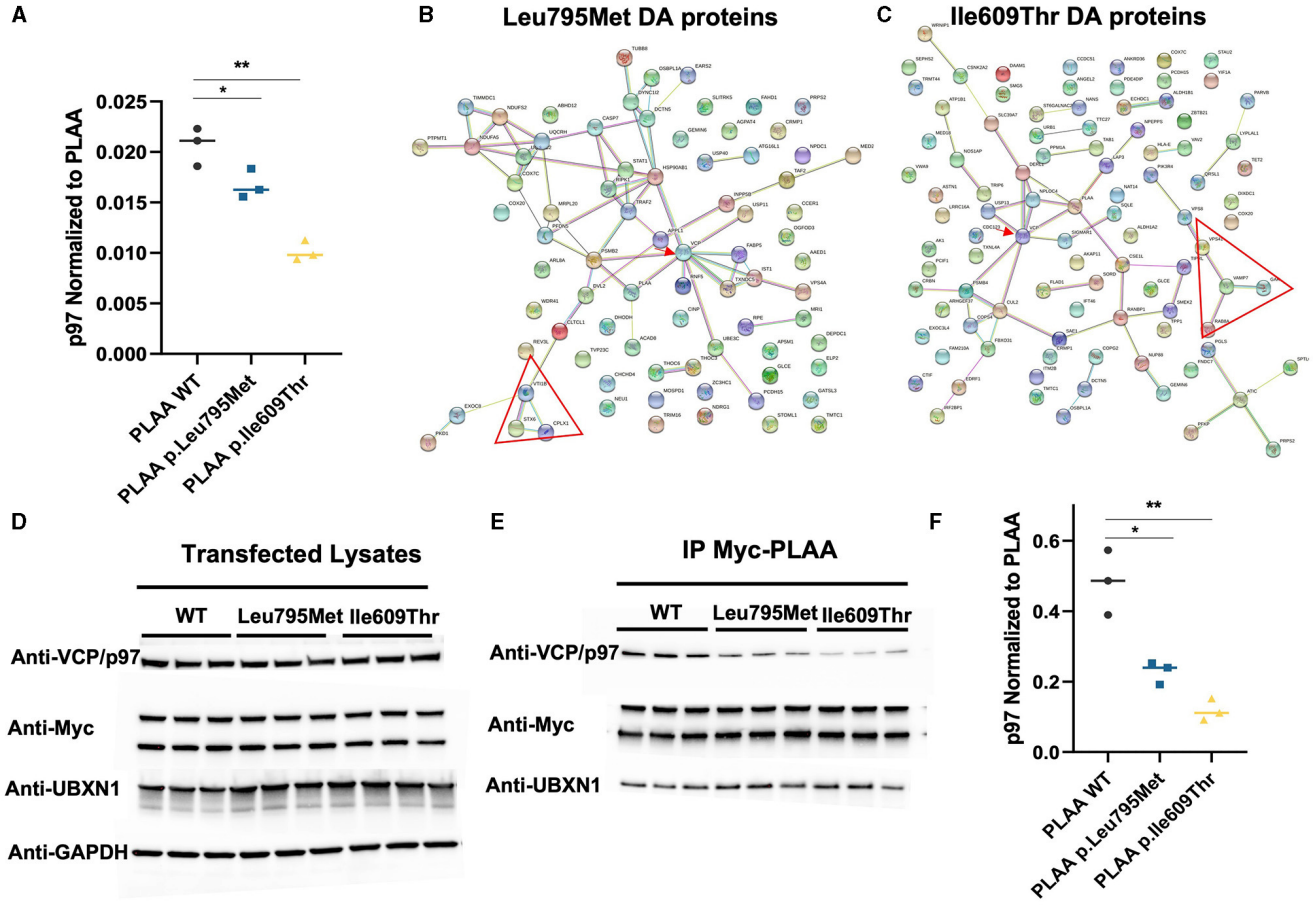
Effect of the two PLAA mutations on the PLAA/p97 interaction. **(A)** Plot of the p97 abundance (as a ratio to PLAA) from the IP Mass Spectrometry data (*p < 0.05, **p < 0.01). **(B, C)** Map of the interaction network of the differentially pulled down proteins in the Leu795Met and Ile609Thr samples compared to wild type. The red arrow indicated VCP/p97. The red triangle indicates a network of SNARE associated proteins **(D)** Western blots of the whole cell lysates from which have undergone immunoprecipitation **(E)** Western blots from the IP elution samples. **(F)** Quantification of p97 from the IP Myc-PLAA elution samples. p97 was divided by the amount of PLAA in the corresponding sample (top Myc-PLAA band) before plotting (*p < 0.05, **p < 0.01).

Considering that both mutations occur in the p97-binding domain of PLAA, we were particularly interested in the effect of the mutations on p97 binding. VCP/p97 was identified in our IP/MS as one of the proteins which was differentially abundant after IP of both p.Leu795Met and p.Ile609Thr compared to WT (Figure 4A). Unexpectedly, this decrease in p97 is more pronounced in the p.Ile609Thr mutant (~50% decrease) as compared to the p.Leu795Met mutant (~20% decrease). In order to get a sense of how the differential binding to p97 affects other PLAA interactions, we mapped the interaction network of the significantly differentially abundant proteins in the p.Leu795Met and p.Ile609Thr IP samples (Figures 4B, C). For both p.Leu795Met and p.Ile609Thr, the PLAA-p97 interaction appear central to the network (red arrow). In particular, known p97-associated proteins such as IST1, FABP5, and PSMB2 for p.Ile609Thr and DERL1, NPLOC4, and SIGMAR1 for Leu795Met were also differentially pulled down by the mutants. Interestingly, for both mutants, we can identify how the identified p.Leu795Met and p.Ile609Thr variants perturbed the PLAA-related interactome based on MS experiments, including a smaller network of SNARE-associated proteins (red triangle) such as namely STX6, VTI1B, and CPLX1 (for the p.Leu795Met mutation) and VAMP7, VPS41, RAB8A, and GAK (for the p.Ile609Thr variant).

We further set out to verify these results using western blot. We first verified that levels of p97 in the total lysates were not affected by the transfections (Figure 4D). We then probed the elution samples with anti-p97 antibody. The western blot shows an even more pronounced decrease in p97 in the p.Leu795Met IP (~50%) compared to WT and in the p.Ile609Thr IP (~80%; Figures 4E, F). We also probed for UBXN1 to verify if the mutation affects binding other p97 cofactors but PLAA-UBXN1 interaction did not seem to be affected by the mutations.

## 4 Discussion

In recent years, studies based on NGS and omics-related sciences revealed an expanding molecular complexity underlying NDDs and ASDs (Ozonoff et al., 2018; Salpietro et al., 2019a,b). The genetic dissection of these conditions is important to correlate the frequent multisystemic and neurological abnormalities to the rare individual neurodevelopmental presentations and fully understand the spectrum associated with different genes (Nicita et al., 2012; Ruggieri et al., 2016b; Salpietro et al., 2018c). Many novel molecular factors have been identified with consequent benefits in terms of refining clinical phenotypes, valuable prognostic information, detailed imaging studies and targeted therapies for the children affected with these conditions (Granata et al., 2016; Baldassari et al., 2020; Donkervoort et al., 2020). Importantly, neurodevelopmental delay, neurodevelopmental regression and ASDs are etiologically highly heterogeneous disorders often co-occurring as the result of distinct monogenic causes (Coleman et al., 2018; Manole et al., 2020; Dworschak et al., 2021; Wiessner et al., 2021). A large clinical study reporting the co-occurrence of regression in ASD among twins suggests several genetic components influencing both classical early onset and regressive ASD phenotypes (Rosenberg et al., 2009). Several molecular studies aimed to dissect the complex architecture of ASD-associated single gene disorders and highlighted frequent deleterious variants in genes often playing important roles in the regulation of synaptic transmission (Salpietro et al., 2018c, 2019a,b; Bell et al., 2019; Dias et al., 2019; Efthymiou et al., 2019; Azzariti and Hamosh, 2020). The human *PLAA* gene (MIM 603873) encodes for a ubiquitin binding protein Phospholipase A2 Activating Protein (PLAP) ortholog of yeast Ufd3/Doa1 (Qiu et al., 2010). Ubiquitination is involved in post-translational modification of specific proteins through adding of ubiquitin (Ub) to lysine residues for more processes (Hall et al., 2017). The Plaa protein composes of two ubiquitin-binding domains and one VCF/p97-binding domain, respectively known as N-terminal WD40 7-bladed propeller-binding, a central PFU domain and a c-terminal PUL (PLAP, Ufd3p, and Lub1p) domain that binds the N-terminal domain of p97 (an AAA ATPase), which transfers ubiquitinated proteins to the proteasome for degradation (Qiu et al., 2010; Hall et al., 2017). The loss of Doa1, in yeast, altering p97/Cdc48 function highlights the importance of the interaction between PLAA and p97 (Qiu et al., 2010). In neurons, alteration of Plaa function disrupts synaptic structure and synaptic vesicle (SV) recycling, involved in impaired synaptic function, suggesting the role of Plaa in ESCRT-mediated endocytic trafficking (Endosomal Sorting Complexes Required for Transport; Hall et al., 2017). The alteration of the *PLAA* gene causes an impairment of turnover and function of synaptic membrane proteins via the endosomal pathway. Presynaptic terminals trafficking is essential for neural function whereby an excess or reduction of SNAREs (e.g., VAMP2 and SNAP25) and other synaptic and membrane proteins, alter synaptic transmission and normal brain developmental processes (Salpietro et al., 2017; Klöckner et al., 2021). To date, only biallelic mutations in *PLAA* gene have been implicated in a Mendelian disease characterized by an early lethal infantile epileptic encephalopathy associated with progressive microcephaly, spasticity, and brain anomalies (NDMSBA) [MIM:617527] (Falik Zaccai et al., 2017; Hall et al., 2017; Dai et al., 2019). In this study, we identified by WES and WGS *de novo* non-synonymous variants affecting highly conserved residues within the PUL domain of PLAA (p.Ile609Thr; p.Leu795Met).

Notably, both affected individuals reported in this study experienced neurodevelopmental regression since late infancy with a tendency of improvement of their language and communication abilities since mid- and late childhood. Based on this observation, it is possible that some of the autistic and other features associated with the *PLAA*-related dominant condition improve with aging. However, at the current stage there are no available long-term follow-up data and/or neurodevelopmental trajectories of adults carrying *de novo PLAA* variants. Of interest, the neurodevelopmental phenotypes described in the affected individuals from this study are reminiscent, although less severe, of the variable neurological phenotypes recently associated with mutations in genes encoding SNAREs. In many of these synaptopathies, usually the recessive diseases are more far more severe than the dominant ones (Salpietro et al., 2017; Lammertse et al., 2020; Maselli et al., 2020). In this study, both the *de novo* variants identified in *PLAA* affected critical residues within the PUL domain. Importantly, the PUL domain is critically involved in the Plaa-p97 interaction and the regulation of Plaa-p97 complex stability and the recycling of synaptic and membrane proteins, including SNAREs (Zhang et al., 2000). Interestingly, the *PLAA* null-mice models revealed an embryonic lethality, in contrast the mice with homozygous missense mutation and compound heterozygous in *PLAA* are born and display early-onset brain developmental and neurodegenerative disorders, highlighting a smaller brain with tremor and motor disorders, including altered gait, hypomotility, and neuromuscular weakness. These traits were indicative of central disturbances in both brain and cerebellar motor circuits (Falik Zaccai et al., 2017; Hall et al., 2017; Dai et al., 2019). The genetic dissection of ASD-related complex molecular architecture is revealing contributions from both coding and non-coding DNA changes (Williams et al., 2019). Deleterious variants in the same genes are often implicated in regressive ASD (Tammimies, 2019) with deleterious variants often implicate genes with important roles of transcriptional and synapse regulation. In this study, no potential environmental factors that could be causal for ASD have been identified. The two *de novo* non-synonymous variants were identified as the only plausible candidate variants to explain the NDD/ASD phenotype of the affected children. However, it is possible that further contributions to the clinical phenotypes result from the effects of modifying genes and additional stochastic processes during development, similar to other genetic NDDs. This may explain potential reduced penetrance associated with monoallelic *PLAA* variants. Importantly, our functional studies using WT and mutant plasmids documented an abnormal Plaa-p97 interaction as a consequence of both variants. The p.Ile609Thr variant has the most severely reduced interaction (~50%) with p97, whereas the p.Leu795Met variant only has 20% reduced interaction. The p.Leu795Met variant was found to alter side chain arrangements at PLAA c-terminal, according to computational simulations. Our study highlights the phenotypic and allelic heterogeneity underlying *PLAA*-related neurological disorders. The identification of abnormal Plaa-p97 interaction as a possible disease mechanism implicates the downstream vesicle recycling in the pathogenesis of dominant *PLAA*-related neurological disorders due to *de novo* mutations affecting the PUL domain.

## Data availability statement

The datasets presented in this study can be found in online repositories. The names of the repository/repositories and accession number(s) can be found at: https://www.uniprot.org/, Q9Y263.

## Ethics statement

The studies involving humans were approved by Gaslini Institutional Review Board. The studies were conducted in accordance with the local legislation and institutional requirements. Written informed consent for participation in this study was provided by the participants' legal guardians/next of kin. Written informed consent was obtained from the individual(s), and minor(s)' legal guardian/next of kin, for the publication of any potentially identifiable images or data included in this article.

## Author contributions

MI: Writing – original draft, Formal analysis. NH: Writing – review & editing, Investigation. SF: Writing – review & editing, Resources, Investigation. JH: Writing – review & editing, Resources, Visualization. SL: Writing – review & editing, Investigation. AR: Writing – review & editing, Resources, Investigation, Resources, Investigation. K-WC: Writing – review & editing, Investigation. IP: Writing – review & editing, Resources, Visualization. AY: Writing – review & editing, Resources, Visualization. SB: Writing – review & editing, Resources, Investigation. ST: Writing – review & editing, Resources, Investigation. GS: Writing – review & editing, Validation. PS: Writing – review & editing, Resources, Investigation. IM: Writing – review & editing, Resources, Investigation. SG: Writing – review & editing, Resources, Investigation. GB: Writing – review & editing, Validation. AC: Writing – review & editing, Validation. DM: Writing – review & editing, Resources. PU: Writing – review & editing, Software. AV: Writing – review & editing, Validation. CF: Writing – review & editing, Validation. AA: Writing – review & editing, Validation. ME: Writing – review & editing, Resources, Visualization. HH: Writing – review & editing, Resources. SS: Writing – review & editing, Resources, Visualization. PS: Writing – review & editing, Supervision. FZ: Writing – review & editing, Funding acquisition, Supervision. T-FC: Writing – review & editing, Conceptualization, Supervision. VS: Writing – review & editing, Conceptualization, Supervision. MS: Writing – review & editing, Resources, Visualization. AD: Writing – review & editing, Validation. SE: Writing – review & editing, Resources. MD: Writing – review & editing, Validation.
