# Supplementary material for: Allelic heterogeneity and abnormal vesicle recycling in PLAA-related neurodevelopmental disorders
Source: Front Mol Neurosci. 2024 Apr 8;17:1268013. doi: 10.3389/fnmol.2024.1268013 (PMC11033462; doi:10.3389/fnmol.2024.1268013)
Supplement: Supplementary file 1 [file Table_1.DOCX]

**SUPPLEMENTARY TABLE 1**

**Table 1. Clinical and genetic heterogeneity of *PLAA*-related neurological disorders**

|  | **This study** | **Falik Zaccai TC *et al*., 2017** | **Hall EA *et al*., 2017** | **Dai C *et al*., 2019** |
| --- | --- | --- | --- | --- |
| **No. of Patients and Gender (M/F)** | 1F/1M | 2F/5M | 2F/8M | 1F/1M |
| **Variant**  **(NM_001031689.3)** | c.2383C>A; WT  c.1826T>C; WT | c.2254C>T | c.68G>T; c.68G>T  c.68dupG; c.68dupG | c.829T>C; c.1049A>T |
| **Mode of inheritance** | *De novo* | Autosomal recessive | Autosomal recessive | Autosomal recessive |
| **Consanguinity** | - | + | + | + |
| **DD/ID** | + | + | + | + |
| **Psychomotor**  **Regression** | + | + | + | NA^°^ |
| **Language skills** | Poor | Poor | Poor | NA^°^ |
| **Behavior (ASD)** | + | - | - | NA^°^ |
| **Seizures** | - | + (3/7) | + (8/10) | + |
| **Postnatal**  **Microcephaly** | - | +* | +^§^ | + |
| **Spasticity** | - | + (7/7) | + (8/10) | + |
| **Leukodystrophy** | - | + | + | + |
| **Other features** | - | pectus carinatum; joints contractures; hirsutism; facial dysmorphisms; optic atrophy | hirsutism; optic atrophy; facial dysmorphisms | pectus carinatum; facial dysmorphisms; |

^*^Until ~5 years of age, followed by a gradual increase in head circumference up to 75% in two cases

^§^One child died too early to be evaluated

^°^Died too early to be evaluated
